# Vaginal microbiome analysis of healthy women during different periods of gestation

**DOI:** 10.1042/BSR20201766

**Published:** 2020-07-24

**Authors:** Dan Li, Xin-Zuo Chi, Lei Zhang, Rui Chen, Jing-rong Cao, Xiao-yan Sun, He-qin Yang, Qin-ping Liao

**Affiliations:** 1 Department of Obstetrics and Gynecology, Xuan Wu Hospital, Capital Medical University, Beijing 100053, China; 2 Department of Obstetrics and Gynecology, Beijing Tsinghua Chang Gung Hospital, Beijing 102218, China; 3 Department of Clinincal Laboratory, Xuan Wu Hospital, Capital Medical University, Beijing 100053, China

**Keywords:** lactobacillus, longitudinal studies, pregnancy, vaginal microbiome

## Abstract

To assess the vaginal microbiome throughout full-term uncomplicated pregnancy, a longitudinal study was designed for 12 healthy women who had prepared to become pregnant and then delivered at term (38–42 weeks) without complications. The vaginal microbial community was studied at pre-pregnancy, 8–12, 24–28, 37–38 weeks of gestation, and puerperium, using hypervariable tag sequencing of the V3–V4 region of the 16S rRNA gene. Sequencing produced approximately 10 million reads on the Illumina MiSeq. Members of the *Firmicutes* phyla were prevailing before and during pregnancy periods, and the proportion was quite as *Proteobacteria* until puerperium. *Lactobacillus* genus was abundant before and during pregnancy, but post-delivery vaginal microflora variety turned diverse. The species-level analysis revealed that a healthy vaginal microbiome before or during pregnancy was prominently dominated by *Lactobacillus crispatus.* Furthermore, PCoA analysis revealed for differences in the bacterial community composition between the two levels of *Lactobacillus* species in pre-pregnancy and pregnancy period (PC1 contribution of 58.46%, PC3 contribution of 8.64%). Based on the taxonomic and PCoA analysis, we found that *L. crispatus* was dominant in the vaginal microflora of healthy women before or during pregnancy, but at the puerperium, the status changed leading to decreased abundance of protective *Lactobacillus* species that made vaginal micro-ecological barrier vulnerable to diseases. Additionally, vaginal pH was an important environmental property affecting the vaginal microbial community.

## Introduction

Local anatomical structure, vaginal flora, immune system, and others collectively create the complex microecosystem of the vaginal microenvironment [1–4]. There are more than 50 kinds of parasitic microorganisms, including *Lactobacillus, Bifidobacterium, Bacteroides, Enterococcus, Staphylococcus epidermidis, Streptococcus, Corynebacterium, Escherichia coli, Vernon coccus, Digestive Streptococcus, Gardnerella, Protozoa, Viruses, Mycoplasma*, and *Candida albicans* that are known to inhabit the vagina of healthy women. Among these, specialized and facultative anaerobes are the dominant ones that flourish in the lateral mucosa of the vagina [5,6]. These microorganisms interact, restrict each other, and remain orderly layered to colonize the vaginal mucosal epithelium by forming the biofilms (BF) [6]. The parasitized vaginal bacteria of BF continue to occur and show varied distribution based on the altered physiological state and the local environment [6]. Among the inhabitants, *Lactobacillus* plays an important role in maintaining the healthy vaginal microecology by preventing the occurrence of vaginal infection [7]. It inhibits the growth of pathogenic microorganisms by producing lactic acid, secreting cytokines, surfactants, H_2_O_2_, and other antimicrobial components [7]. Besides, using competitive adhesion mechanism, it also prevents pathogenic microorganisms from adhering to vaginal epithelial cells and stimulates the immune system to maintain the vaginal microecological balance.

The vaginal microecological environment is a unique dynamic system that is constantly changing depending upon the local physiological state and chemical factors. These influencing factors can be broadly divided as endogenous and exogenous based on their nature. The level of hormone estrogen also plays a decisive role. Several studies found that various factors such as menstruation, vaginal irrigation, contraceptives, vaginal medication, number of sexual partners, frequency of sexual intercourse, and the use of hygienic products (sanitary napkins or tampons) during menstruation are capable of altering the composition of the vaginal flora. During the pregnancy, elevated estrogen level facilitates *Lactobacillus* to occupy a dominant position in vaginal flora, however, at the same time, the incidence of *Vulvovaginal candidiasis* also turns higher [8–10]. The secretions from the vaginal vestibular glands increases and the vulva remain in a wet state. This scenario is highly conducive for the growth of *Lactobacillus, Staphylococcus epidermidis, Candida, Enterococcus faecalis, Propionibacillus, Corynebacter*, and *Mycoplasma hominis.* In addition, increased vaginal mucosa congestion, edema, and permeability also make vaginal mucosa more vulnerable to injury than that of a pre-pregnancy period. Therefore, pregnant women become more prone to various vaginal infections. The multiple scientific reports suggest that the pregnancy infection(s) can result in adverse pregnancy outcomes such as spontaneous abortion, premature delivery, premature rupture of membranes, amniotic fluid infection, chorioamnionitis, postpartum endometritis, soft birth canal laceration and so on [11–16].

Most vaginal microecology studies majorly focused on the premature delivery and premature rupture of membranes to improve understanding for the clinical applications but these targeted the specific periods of pregnancy [17]. There are very few studies, especially in women of the yellow race in China, for the complete pregnancy period, a longitudinal study that also included the pre-pregnancy period. The dynamic observations from such a study will significantly improve the understanding of the vaginal microflora in maintaining normal physiology throughout the pregnancy. The present study specifically aims to answer the aforesaid query. The findings are valuable to prevent reproductive tract infection and in reducing the adverse pregnancy outcomes.

## Methods

### Recruitment of subjects

A total of 350 women with recent pregnancy were selected in the outpatient Department of Gynecology of Xuanwu Hospital of Capital Medical University from October 2013 to October 2015. Of these 12 cases of healthy women, aged 31.5 ± 5.5 years were enrolled for the MiSeq sequencing analysis in different gestational phases. The following subjects were excluded from the study: those who used vaginal medication for any reason within 1 week; those who used antibiotics or antifungals for any reason within 1 week; those who had any of the symptoms or similar to pruritus, burning, leukorrhea abnormalities, urinary tract infection, and diarrhea within 2 weeks; those have been diagnosed with any of the following diseases within 6 months, including pelvic inflammatory disease, cervicitis, vaginitis, other gynecological inflammatory diseases (mycoplasma, chlamydia, gonorrhea, syphilis, etc.); if the sexual partners had recently been diagnosed with sexually transmitted diseases (mycoplasma, Chlamydia, gonorrhea, syphilis, etc.); those who had positive signs in the gynecological examination; vaginal discharge with blood; sex partners might be infected with chlamydia or have an anorectal chlamydia infection or have another partner with a sexually transmitted infections (STIs), requiring different treatment. Women were enrolled in the trial only once and might have participated in other non-conflicting research projects. Additionally, women were not selected if they underwent either spontaneous or indicated preterm delivery before 37 weeks, or if they developed any intercurrent infection requiring antibiotic therapy.

The study started with voluntary enrollment of 350 women with recent pregnancy. After primary selection, only 150 of these were enrolled. From these too, 80 women were further recruited to a longitudinal study at booking of their antenatal care. The vaginal swab samples were collected at the initial visit before pregnancy, at gestational ages 8–12, 24–28, and 37–38 weeks and then 6 weeks after the child delivery. Out of these, vaginal samples from 20 women showed normal Gram staining results. From these too, only a few pregnant women (*n*=12) with an uncomplicated singleton pregnancy had no medical problems or adverse outcomes during any previous pregnancy. The study had approval from the Ethical Committee and written informed consent was obtained from the parturient. All experiments were conducted under the approved guidelines.

### Collection of samples

Only a few pregnant women (*n*=12) had uncomplicated singleton pregnancy and no medical problems or adverse outcomes during any previous pregnancy. Among these patients, two swabs of vaginal secretions were taken from the upper one-third lateral wall of the vaginal vault at the clinic. One sample was used for Gram staining and the other was used for the bacterial genomic DNA extraction. Samples were collected at the initial visit before pregnancy, at gestational ages 8–12, 24–28, and 37–38 weeks and then 6 weeks post-delivery. The first swabs were stored in 1 ml of PBS and frozen on dry ice for transportation or to store at −80°C. The first vaginal swab of vaginal fluid was used for genomic DNA extraction. Using second vaginal swabs, based on the morphological observations of the Gram staining slides, vaginal infection status was determined. The diagnostic criteria were according to a previously published literature [18]. Detailed medical and gynecological history of subjects was obtained from the electronic medical record system of the hospital.

### DNA extraction and PCR amplification

TIANamp Bacteria DNA Extraction Kit (Tiangen Biotech, Beijing, China) was used for the extraction of microbial DNA from the vaginal specimens as per manufacturer’s protocols. The V3–V4 region of the bacteria 16S rRNA genes were amplified using PCR (95°C for 3 min, followed by 30 cycles at 98°C for 20 s, 58°C for 15 s, and 72°C for 20 s and a final extension at 72°C for 5 min). The reactions were performed in 30 μl mixture containing 15 μl of 2× KAPA Library Amplification ReadyMix, 1 μl of each 338F and 806R primers (10 μM), 50 ng of template DNA, and ddH_2_O. The sequences of the primers used were as follows: 338F 5′- ACTCCTACGGGAGGCAGCAG-3′ and 806R 5′-GACTACHVGGGTWTCTAAT-3′.

### MiSeq sequencing

Amplicons were gel purified from 2% agarose gels using the AxyPrep DNA Gel Extraction Kit (Axygen Biosciences, U.S.A.) as per the manufacturer’s instructions. The sample DNA concentrations were quantified using Qubit®2.0 (Invitrogen, U.S.A.). The prepared library was sequenced using tags on the MiSeq platform (Illumina Inc., U.S.A.) for paired-end reads of 250 bp. It approximately produced 10 million reads. These were overlapped on their 3′ ends for concatenation into original longer tags. DNA extraction, library construction, and sequencing were conducted at the Realbio Genomics Institute (Shanghai, China).

### Process of sequencing data

Quality of tags, trimmed barcodes, and primers were checked for the lengths and average base quality. The length of 16S tags was restricted to 220 and 500 bp so that the average Phred score of bases was no worse than 20 (Q20) and no more than three ambiguous N. The copy number of tags was enumerated and redundancy of repeated tags was removed. Only the tags with a frequency of more than 1 were considered reliable to cluster into Operational Taxonomic Units (OTUs). Each of these had a representative tag. OTUs were clustered with 97% similarity using UPARSE (http://drive5.com/uparse/) and chimeric sequences were identified and removed using USEARCH (version 7.0). RDP Classifier (http://rdp.cme.msu.edu/) was used to assign taxa to each representative tags against the RDP database (http://rdp.cme.msu.edu/) based on the confidence threshold of 0.8. OTU profiling table and α/β diversity analyses were carried out using python scripts of Qiime.

### Statistical analyses

Statistical analysis was carried out using SPSS 23 (SPSS Inc., Chicago, IL, U.S.A.) program. Continuous data are expressed as the mean ± standard deviation. Categorical data are expressed as frequencies. For statistical analysis of differences in vaginal pH, we used the Friedman’s test followed by Dunn’s post-test. Data were considered significantly different when *P*<0.05.

## Results

### Characteristics of subjects

**Figure 1:**
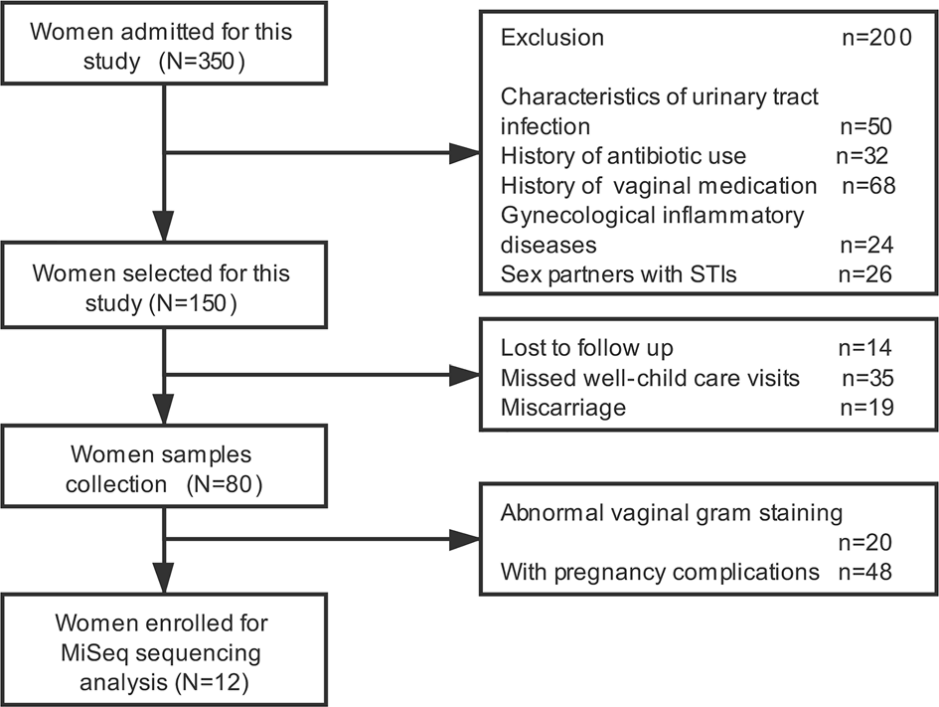
The flow chart of patient recruitment in the present study

Initially 350 women volunteered to participate in the study. A series of selection criteria were employed to finally select 12 pregnant women to carry out the research. The detailed selection process is summarized in Figure 1 and the clinical and demographic characteristics of the 12 finally selected subjects are displayed in Table 1. The average age of these women was 31.5 ± 5.5 years. 16S rRNA high throughput sequencing samples were collected at various stages of pregnancy. The specimens were collected on average 8.3 ± 1.4 weeks before pregnancy. Samples during pregnancy were collected in the first trimester, second trimester, and in the third trimester of pregnancy. These had an average time of 10.3 ± 1.6, 24.6 ±1.3, and 38.1 ± 1.1 weeks, respectively. Post-delivery samples were collected after 6.4 ±1.3 weeks of age.

**Table 1:** Descriptive characteristics of the pregnant woman enrolled in the longitudinal study (*n*=12)

	Mean/Cases (percentage)	SD	Minimum	Maximum
Age (years)	31.5	5.5	24	42
Prepare time (weeks)	8.3	1.4		
Early pregnancy (weeks)	10.3	1.6		
Middle pregnancy (weeks)	24.6	1.3		
Late pregnancy (weeks)	38.1	1.1		
After delivery time (weeks)	6.4	1.3		
Body mass index (BMI; kg/m^2^)	33.4	4.6	27.8	44.7
Nulliparity	11 (91.7%)			
Cesarean delivery	8/12 (66.7%)			
Gestation age at delivery (weeks)	39.6	1.2		
Birth weight (grams)	3345	523	2950	4250
Apgar at 1 min (median)	9.9		9	10
Apgar at 5 min (median)	10		10	10

### Characteristics of the species during the normal pregnancy

According to OTU criteria, reads in an OTU are highly likely to be sampled from the similar species. The samples from the before and during pregnancy revealed the abundance of the species from genus *Lactobacillus* and other bacteria. However, *Lactobacillus* was in higher abundance and majorly included *L. crispatus, L. gasseri, L. jensenii, L. johnsonii*, pentose-utilizing *Lactobacillus, L. reuteri, L. plantarum, L. rhamnosus, L. helveticus, L. acidophilus, L. brevis*, vaginal *Lactobacillus*, inert *Lactobacillus*, rat’s *Lactobacillus, L. casei, L. kunkeei*, a group of unclassified *Lactobacillus*, and others. A total of 19 kinds of *Lactobacillus* species were identified and there are summarized in Table 2.

**Table 2:** Core microbiome

Level	name
genus	Lactobacillus
species	Lactobacillus_acidophilus
species	Lactobacillus_brevisF
species	Lactobacillus_crispatus
species	Lactobacillus_delbrueckii
species	Lactobacillus_fermentans
species	Lactobacillus_gasseri
species	Lactobacillus_helveticus
species	Lactobacillus_iners_AB-1
species	Lactobacillus_jensenii
species	Lactobacillus_johnsonii
species	Lactobacillus_kunkeei
species	Lactobacillus_murinus
species	Lactobacillus_pentosus
species	Lactobacillus_plantarum
species	Lactobacillus_reuteri_JCM_1112
species	Lactobacillus_rhamnosus
species	Lactobacillus_sakei
species	Lactobacillus_salivariusF
species	Lactobacillus_vaginalis
species	Lactobacillus_unclassified

### Phylum level analysis of vaginal microbiota

**Figure 2:**
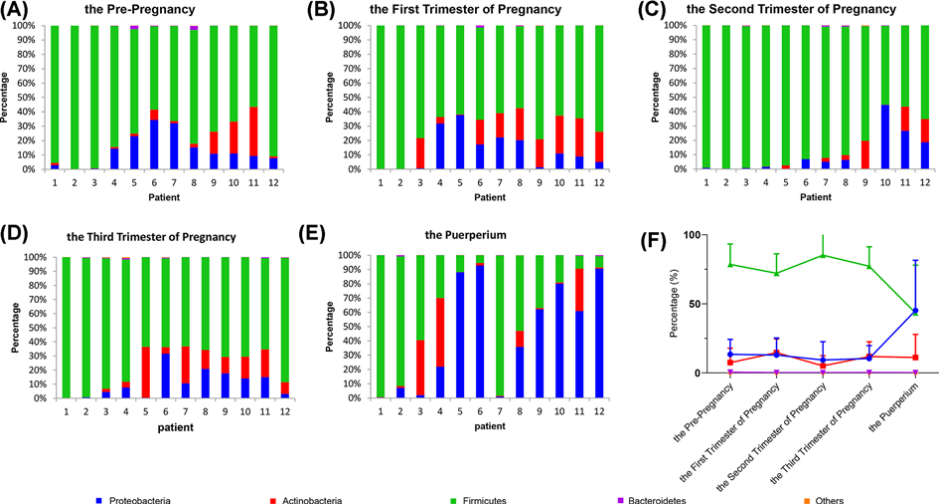
Phylum level analysis of vaginal microbiota (**A**–**E**) Twelve samples mainly include five taxonomic compositions of vaginal bacterial communities before pregnancy and in the three trimesters of pregnancy and at the puerperium. (**F**) Line graph represents mean ± SEM from the 12 independent data.

Among the 12 selected women samples, we found that the *Firmicutes* account for the highest proportion of the vaginal bacteria before and during pregnancy, followed by the *Proteobacteria, Actinobacteria, Bacteroidetes*, and then lastly a small group of other bacterial population (Figure 2A–D,F). However, in post-pregnancy (puerperium) samples, the population of *Firmicutes* decreased sharply, while the *Proteobacteria* increased even as much that they now matched the percentage of the *Firmicutes* (Figure 2E,F).

### Genus level analysis of the vaginal microbiota

**Figure 3:**
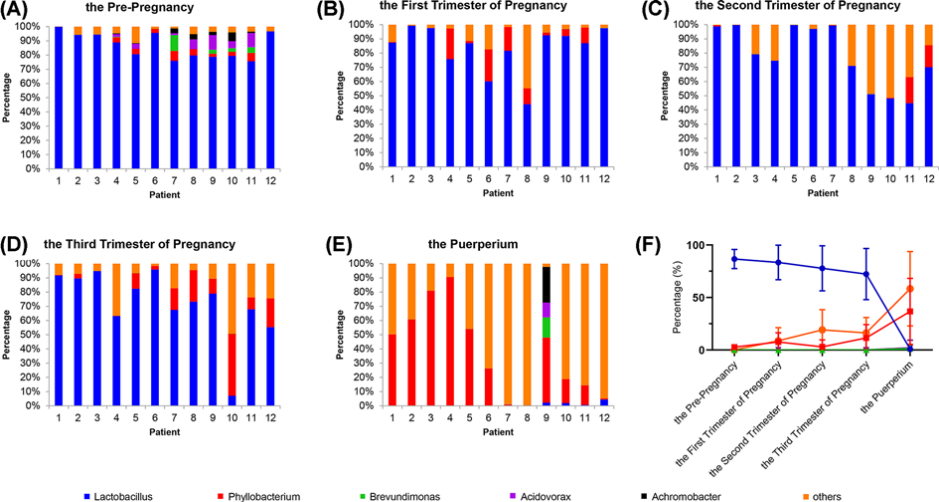
Genus level analysis of the vaginal microbiota At a lower taxonomic level, the five most-abundant Lactobacillaceae families were shown and ‘Others’ denoted the less abundant. (**A**–**E**) Representative data of 12 healthy women before pregnancy and in the three trimesters of pregnancy and at the puerperium is shown. (**F**) The change of composition is also presented as a line graph.

At a lower taxonomic level, Lactobacilli were predominant in healthy women before or during pregnancy, but the percentage gradually decreased from 86.6% of the pre-pregnancy period to 72.3% at the third trimester of pregnancy (Figure 3A–D,F). At the puerperium stage, *Lactobacillus* abundance significantly reduced to 0.83% while *Phyllobacterium* increased to 36.7% from 2.7% of the pre-pregnancy period (Figure 3E,F). Besides, the occurrence of other types of vaginal bacteria such as *Lactobacillus, Phyllobacterium, Brevundimonas, Acidovorax*, and *Achromobacter*, excluding those mentioned in Figure 3 improved during post-pregnancy, compared with the before or during pregnancy periods that majorly included *Bacillus, Pseudomonas, Streptococcus, Prevotella, Lysinibacillus*, and *Oceanobacillus* (data not shown).

### Species level analysis of the vaginal microbiota

**Figure 4:**
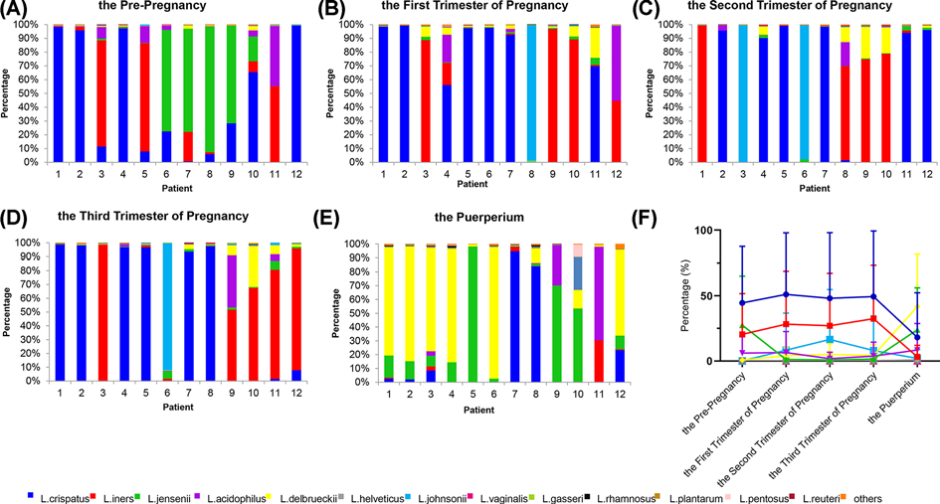
Species level characterization of *Lactobacillus* genus The 13 most-abundant species are shown and ‘Others’ denoted the less abundant *Lactobacillus* species. (**A–E**) represents the relative genus abundance of 12 women before pregnancy and in the three trimesters of pregnancy and at the puerperium and (**F**) the average is applied to produce a line chart.

Since *Lactobacillus* is vital in maintaining the vaginal microenvironment, a species level analysis on this genus was performed (Figure 4). Here, we found that among *Lactobacillus, L. crispatus* dominated for over 40% of the total *Lactobacillus* population in the vaginal microbiota of healthy subjects while a shift toward to a significantly lower level can be noticed in the post-pregnancy period. *L. iners* are the second highest with approximately 30% abundance and showed a change similar to *L. crispatus* (Figure 4A–D,F). On the contrary, *L. johnsonii* showed a significant increase only in the second trimester of the pregnancy. Interestingly, in post-pregnancy samples, *L. delbrueckii* and *L. jensenii* with respective abundance of 41.8 and 24.0% dominate over *L. crispatus* which now has decreased to 18.1% and other bacterial species (Figure 4E,F). Importantly, the pre-pregnancy abundance of *L. delbrueckii* was merely 0.8% and had an average abundance of only 4.5% during the complete pregnancy period.

### PCoA analysis of vaginal microbiome

**Figure 5:**
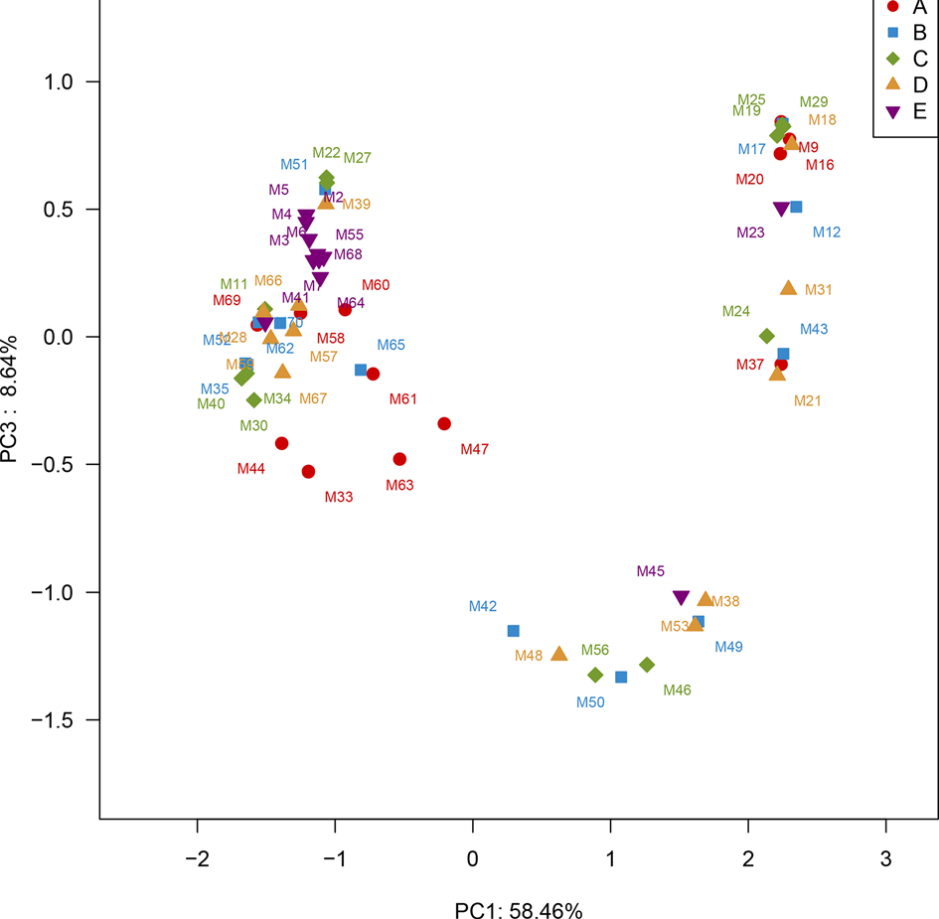
PCoA of vaginal microbiome Principal Coordinates Analysis (PCoA) on Bray–Curtis distance showing the separation of the samples from healthy women’s vagina before, during pregnancy and after delivery is shown. (**A**) Pre-pregnancy period; (**B**) the first trimester of pregnancy; (**C**) the second trimester of pregnancy; (**D**) the third trimester of pregnancy; (**E**) puerperium period. M: A symbol with no special meaning. The number followed by M indicates the encoding of each sample.

**Figure 6:**
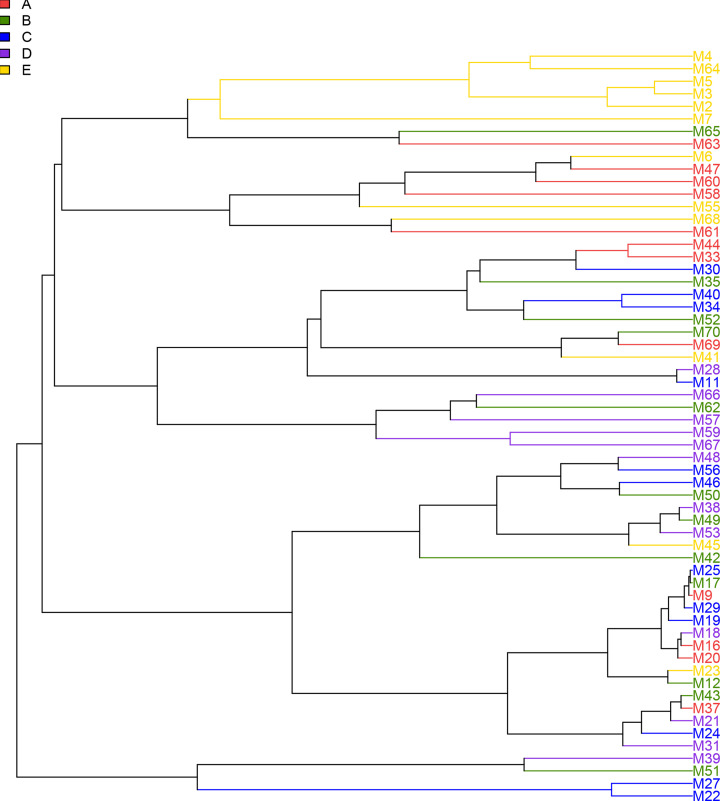
Cluster analysis chart Red represents the prepared pregnancy, green represents the first trimester of pregnancy, blue represents the second trimester of pregnancy, purple represents the third trimester of pregnancy, yellow represents the puerperal period. (**A**) pre-pregnancy period; (**B**) the first trimester of pregnancy; (**C**) the second trimester of pregnancy; (**D**) the third trimester of pregnancy; (**E**) puerperium period. M: A symbol with no special meaning. The number followed by M indicates the encoding of each sample.

In the PCoA diagram, OTU distribution in different pregnancy periods is shown (Figure 5). The color codes are as following: red represents the pre-pregnancy, green represents the first trimester of pregnancy, blue represents the second trimester of pregnancy, purple represents the third trimester of pregnancy, yellow represents the puerperal period. The sample differences contribution rate is 58.46%, the vertical axis represents the second principal components of the sample difference where the contribution rate is 8.64%. It effectively puts apart the pre-pregnancy and puerperium vaginal bacteria community composition, but could not distinguish the early, middle, and late pregnancy periods. Furthermore, based on Unifra distance a UPGMA (Unweighted Pair Group Method with Arithmetic Mean) cluster tree was created to assess the similarity of bacterial community composition among all samples. Here, sample-weighted clustering analysis could effectively cluster pre-pregnancy and puerperal samples but not the early, middle, and late pregnancy samples (Figure 6).

### The vaginal pH value during the normal pregnancy

The vaginal pH was 4.06 ± 0.19, 3.84 ± 0.05, 3.88 ± 0.08, 3.88 ± 0.15, and 5.14 ± 0.10 in the pre-pregnancy period, the first trimester of pregnancy, the second trimester of pregnancy, the third trimester of pregnancy and puerperium stage, respectively. There was no statistically significant difference in vaginal pH among the first trimester of pregnancy, the second trimester of pregnancy, and the third trimester of pregnancy. However, the vaginal pH in the pre-pregnancy period and puerperium stage were markedly higher than those in the the first trimester of pregnancy, the second trimester of pregnancy, and the third trimester of pregnancy (*P*<0.01). Moreover, the vaginal pH in puerperium stage were significantly higher than that in the pre-pregnancy period (*P*<0.01).

## Discussion

The vaginal microecological environment is affected by multiple factors and gets substantially altered during pregnancy [18]. Traditionally, vaginal microflora samples were studied using bacterial culture methods [19]. However, in this technique a substantial population of the microorganisms could not be detected due to the limitation of conditioned culture under laboratory conditions [20]. For instance, *L. inertia*, a common type of vaginal bacteria does not grow on conventional selective *Lactobacillus* media (MRS medium and Rogosa medium) [14]. Compared with the traditional microbial culture techniques, 16S rRNA high-throughput sequencing provides more comprehensive information on bacterial community structure. This method is also advantageous due to high reproducibility, reliability, and speed. Moreover, it can detect multiple samples simultaneously [21]. It is a typical molecular biology based exercise that is largely independent of traditional culture methods. Technology-driven 16S rRNA high-throughput sequencing method is also much more comprehensive in exploring the diversity and complexity of vaginal microbial colonies, as well as the differences of vaginal microbial community composition in different diseases and physiological states [22].

The majority of previous studies reported the difference of vaginal microflora between the healthy pregnant women and those with either vaginal infection or other complexed complications [23–25]. But in this study, using the high-throughput sequencing analysis technique, we reported the composition of vaginal flora during the different periods of gestation in healthy childbearing aged women of Han nationality in northern China. In addition, the PCoA and Unifra-based UPGMA mean clustering analysis methods were employed to compare the total vagina bacterial community in the respective samples.

The high-throughput sequencing analysis revealed the existence of a large number of bacterial communities that showed unique microflora characteristics and diversity. We found the distinct distribution of various bacterial species depending on the period of pregnancy. *L. crispatus, L. iners, L. jensenii*, and *L. acidophilus* were the top four strains of *Lactobacillus* in the early pregnancy and showed relatively high abundance. This is also in accordance with a previously published study that also reported these as the most common bacterial species in the vagina of healthy women of childbearing age [26]. Though the composition and diversity of the vaginal-associated bacterial community in healthy people have distinct distribution characteristics, *Lactobacillus* is still the dominant species. It is considered as the most stable part of vaginal microflora [27,28]. It is also suggested that among the four major *Lactobacillus* strains, *L. crispatus* is most stable and does not readily change during different stages of pregnancy whereas the stability of *L. jensenii* is in the middle, and *L. gasseri* and *L. inertia* shows the least stability [27]. In this study too, we detected the uniform presence of *L. crispatus* in several samples with a high detection rate. This again suggests that *L. crispatus* has the strongest adaptability and viability that help in maintaining the regular vaginal microflora. Since *L. acidophilus* is a group of substantially genetically diverse species with deep phylogenetic branches, the proportion of the specific bacterial species in the vaginal microflora can substantially change with countries, regions, and races. This highlights the need for vaginal microflora studies in different ethnic groups in distinct regions of China that could have a diverse proportion of the bacterial species.

There are significant differences in the composition and diversity of vaginal microflora between healthy women of childbearing age and pregnant women. This suggests that during pregnancy vaginal microflora plays an important role in the host [29]. The composition and diversity of bacterial communities in the vagina during early and late pregnancy are usually similar. However, post-pregnancy dynamic changes take place due to the altered level of estrogen, glycogen, lactic acid, and pH in the vaginal microenvironment [28,29]. Our study showed that vaginal pH was important environmental properties affecting the vaginal microbial community.

Multiple countries study shows that *Lactobacillus* is the dominant bacteria and the species composition is relatively constant during normal pregnancy [10,30]. However, post-delivery, *Lactobacillus* gets replaced by *Phyllobacterium, Bacillus, Pseudomonas, Streptococcus, and Prevotella*. These changes could be the result of fluctuation in estrogen level, the trauma of delivery, and vaginal operation [30]. The low estrogen levels during the postpartum decrease the *L. vaginalis* abundance that is another *Lactobacillus* species which is part of healthy vaginal microflora. This increases the vaginal vulnerability to conditional pathogens, thereby increasing the chance of infection [30]. Moreover, vaginal dryness and atrophy are also associated with the down-regulation of genes that are related to the maintenance of epithelial cell structure, barrier function, and up-regulation of inflammation-related genes [31]. Also, in lochia, vaginal natural protection mechanism gets compromised, vaginal pH value increases and the environment for the growth of *Lactobacillus* turns unfavorable. This further reduces its abundance. It is recommended that *Lactobacillus* probiotic supplement during puerperium can substantially improve the vaginal microenvironment and reduce puerperium infection [30]. Overall, it is vital to maintain the vaginal micro-ecological balance to prevent the reproductive tract infection and adverse outcome during the pregnancy by maintaining the dominant position of the beneficial *Lactobacillus*.

Nonetheless, this study has a few limitations due to the comparatively lower number of test subjects, restricted to a single-center. It mainly focuses on the healthy women. Therefore, in the future, a multi-center study with a larger sample size is necessary to evaluate the vaginal microflora of pregnant women in the overall population. This might also help in decreasing the potential selection bias if any. Additionally, vaginal flora changes has an impact on pregnancy complications. However, the sample size is not large enough to see the role of vaginal microflora change in the complication in pregnancy. Further studies were still needed to investigate and compare the vaginal microflora of the pregnant women vs complicated pregnancy.

In conclusion, this study showed a huge change in the vaginal microbial population during different periods of pregnancy and the Puerperium. These findings lay the foundation for analyzing and searching for the composition ratio of bacteria that are more beneficial to pregnancy outcomes, and also provide a basis for screening and developing probiotics suitable for women's reproductive tract health in China.

## Abbreviations

BF: biofilms
OTU: Operational Taxonomic Unit
UPGMA: unweighted pair group method with arithmetic
