# Correction: Vaginal microbiome analysis of healthy women during different periods of gestation

**DOI:** 10.1042/BSR-20201766_COR

**Published:** 2020-09-29

**Keywords:** lactobacillus, longitudinal studies, pregnancy, vaginal microbiome

The authors of the original paper “Vaginal microbiome analysis of healthy women during different periods of gestation” (*Biosci Rep*. (2020), 40(7), DOI: 10.1042/BSR20201766), would like to correct the following statement from the last paragraph of the introduction in their paper:

“There are very few studies, especially in women of the yellow race in China, for the complete pregnancy period, a longitudinal study that also included the pre-pregnancy period.”

Which should have read;

“There are very few studies, especially in women of the Han nationality in China, for the complete pregnancy period, a longitudinal study that also included the pre-pregnancy period.”

The Journal and the authors apologise for this error and for any offence caused.

